# Molecular Insights into Poly(ADP-ribose) Recognition and Processing

**DOI:** 10.3390/biom3010001

**Published:** 2012-12-21

**Authors:** Roko Žaja, Andreja Mikoč, Eva Barkauskaite, Ivan Ahel

**Affiliations:** 1 Cancer Research UK, Paterson Institute for Cancer Research, University of Manchester, Wilmslow Road, Manchester M20 4BX, UK; E-Mails: rzaja@irb.hr (R.Z.); EBarkauskaite@picr.man.ac.uk (E.B.); 2 Rudjer Boskovic Institute, Bijenicka 54, Zagreb 10000, Croatia; E-Mail: mikoc@irb.hr (A.M.)

**Keywords:** Poly(ADP-ribose), PARP, PARG, macrodomain, protein modification

## Abstract

Poly(ADP-ribosyl)ation is a post-translational protein modification involved in the regulation of important cellular functions including DNA repair, transcription, mitosis and apoptosis. The amount of poly(ADP-ribosyl)ation (PAR) in cells reflects the balance of synthesis, mediated by the PARP protein family, and degradation, which is catalyzed by a glycohydrolase, PARG. Many of the proteins mediating PAR metabolism possess specialised high affinity PAR-binding modules that allow the efficient sensing or processing of the PAR signal. The identification of four such PAR-binding modules and the characterization of a number of proteins utilising these elements during the last decade has provided important insights into how PAR regulates different cellular activities. The macrodomain represents a unique PAR-binding module which is, in some instances, known to possess enzymatic activity on ADP-ribose derivatives (in addition to PAR-binding). The most recently discovered example for this is the PARG protein, and several available PARG structures have provided an understanding into how the PARG macrodomain evolved into a major enzyme that maintains PAR homeostasis in living cells.

## 1. Metabolism and Cellular Function of PAR

**Figure 1:**
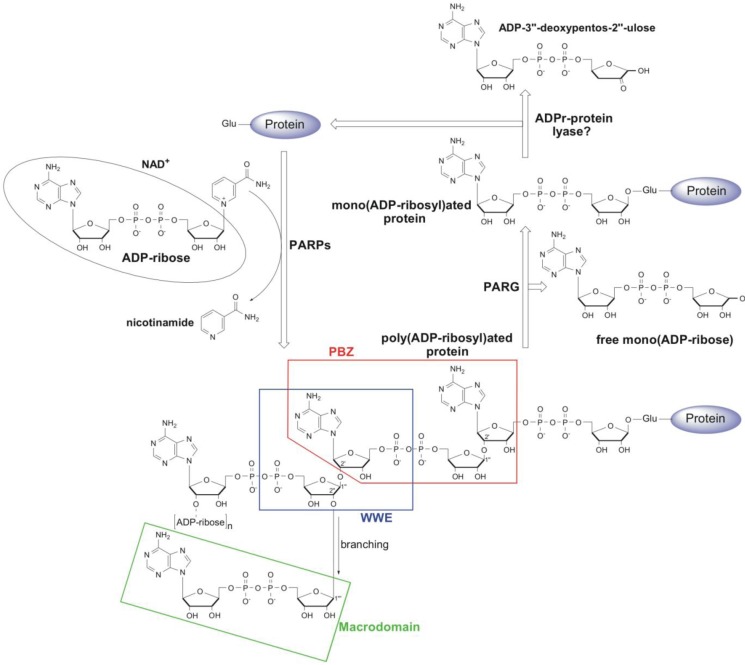
Schematic representation of poly(ADP-ribosyl)ation (PAR) metabolism. PARPs catalyse the transfer of an ADP-ribose moiety from NAD+ to a target protein with the concomitant release of nicotinamide. The first ADP-ribose is attached via an ester linkage to the glutamate on the target protein. The subsequent transfer of additional ADP-ribose molecules through the 2',1''- and 2'',1'''-*O*-glycosidic bond leads to the synthesis of linear and branched PAR polymers, respectively. PARG cleaves PAR chains and releases mono(ADP-ribose), whilst the proximal ADP-ribose is removed by ADP-ribosyl protein lyase. Regions of PAR recognised by Poly(ADP-Ribose)-Binding Zinc Finger (PBZ), WWE and macrodomain PAR-binding modules are boxed.

Protein ADP-ribosylation is a post-translational modification in which an ADP-ribose moiety from NAD^+^ is transferred to a target protein, with the concomitant release of nicotinamide (Figure 1). This reaction is mainly catalysed by members of the ADP-ribosyl transferase (ART) superfamily. The covalent attachment of one ADP-ribose leads to mono(ADP-ribosyl)ation while the subsequent transfer of additional ADP-ribose molecules through *O*-glycosidic ribose-ribose bonds results in the synthesis of poly(ADP-ribose) (PAR) polymers [1]. The ART superfamily contains two major protein families involved in protein ADP-ribosylation, ecto-ARTs and poly(ADP-ribosyl) polymerases (PARPs). The ecto-ARTs exclusively catalyse mono(ADP-ribosyl)ation and were first discovered in bacterial toxins including cholera, pertussis, diphtheria and botulinum toxin [2,3]. The mammalian ecto-ARTs (mART1-5) are extracellular mono(ADP-ribosyl) transferases that share low sequence similarity to toxins, but are structurally and functionally conserved [4,5]. The intracellular enzyme counterparts of ecto-ARTs in mammals are as yet to be identified [5,6]. The poly(ADP-ribose) polymerase (PARP) family of proteins represents a unique subgroup of ARTs because some of its members are able to catalyse poly(ADP-ribosyl)ation. The poly(ADP-ribosyl)ation and PARP family are inherently present only in eukaryotes (with the exception of yeast), whilst prokaryotes and archaea were traditionally thought to be devoid of the poly(ADP-ribosyl)ation machinery [1,7,8,9]. It was recently shown however, that some bacterial species do indeed possess PARP proteins that are highly homologous to the vertebrate PARPs. It is likely that these genes were acquired through horizontal gene transfer [1,10]. To date 17 members of the PARP family have been identified in humans where they regulate many important cellular functions such as DNA repair, transcription, regulation of centromere function, telomere length and ageing, protein degradation, apoptosis and necrosis [11,12,13]. Based on their structural and catalytic properties they can be divided into three main groups. PARPs 7–8, 10–12 and 14–16 are thought to be only capable of mono(ADP-ribosyl)ation, whilst PARP9 and PARP13 lack several conserved amino acid residues that are implicated in NAD-binding in other family members and are therefore thought to be catalytically inactive [9,14,15]. PARP members 1–6 are the only PARPs whose catalytic triads (H-Y-E) contain the highly conserved glutamate residues that have been shown to be critical for the ability to synthesise PAR [13]. PARPs covalently attach to the first ADP-ribose unit through an ester bond to the carboxyl group of the glutamate or aspartate residues on target proteins [16,17]. Recent studies have suggested that lysine residues could also act as acceptors in the poly(ADP-ribosyl)ation reactions [18,19]. The subsequent transfer of additional ADP-ribose molecules through 2',1''-*O*-glycosidic ribose-ribose bonds leads to the synthesis of linear PAR polymers. In addition, a branching reaction has been suggested to occasionally occur through the 2'',1'''-glycosidic bond, resulting in a highly complex network of PAR polymers consisting of up to 200 ADP-ribose subunits [20,21,22,23] (Figure 1).

Poly(ADP-ribosyl)ation has been most extensively studied in the regulation of DNA repair. Three DNA break inducible PARPs (PARP1, PARP2 and PARP3) are involved in these processes [24,25]. The PAR levels in cells are usually low, but in response to DNA damage, DNA repair PARPs are activated and this ultimately leads to the rapid synthesis of PAR chains on target proteins [26]. A number of proteins are known to be PARylated in response to DNA damage (e.g., XRCC1, p53, histones), and the most robust poly(ADP-ribosyl)ation upon genotoxic stress is on PARP1 itself (automodification) [27,28]. PAR modification controls several important aspects of DNA repair. Firstly, it acts as a recruitment scaffold for core DNA repair factors, such as XRCC1 and DNA ligase III [29,30]. Furthermore, chromatin remodelers such as ALC1 and CHD4 and the histone chaperone APLF (aprataxin PNK-like factor) are also recruited to DNA damage sites through PAR binding, where they modulate chromatin structure during the DNA repair process [31,32,33]. Finally, PAR can also act as a major apoptotic signal [34]. In addition to damaged DNA, another major site of poly(ADP-ribosyl)ation is the mitotic apparatus (mitotic spindle, centromeres and centrosome) where poly(ADP-ribosyl)ation is mediated by tankyrase 1 (PARP5a) [35,36,37]. Tankyrase 1 and 2 (PARP5a/b) are also known to be involved in telomere homoeostasis and integrity [38,39]. Poly(ADP-ribosyl)ation can also regulate the cell cycle progression through the function of the mitotic checkpoint protein CHFR (checkpoint protein with FHA and RING domains) [40,41]. In fungi, poly(ADP-ribosyl)ation has a role in replicative ageing [42,43].

In accordance with its cellular significance, poly(ADP-ribosyl)ation is a highly dynamic and reversible process that must be tightly controlled. It has been shown that most of the PAR generated upon genotoxic stimuli is rapidly degraded, with a half-life of 40 s to 6 min [44]. This efficient PAR turnover is mediated by poly(ADP-ribose) glycohydrolase (PARG). PARG hydrolytically cleaves PAR chains and releases mono(ADP-ribose) as the main reaction product [10,45]. The crystal structure of five PARG homologues was recently solved and the precise molecular mechanisms of how PARGs recognize and degrade the PAR chains will be addressed in detail later in this review [10,46,47] (PDB IDs: *M. musculus* PARG (4FC2), human PARG (4AOD), *Tetrahymena thermophila* (4EPP), *Thermomonospora curvata* (3SIG) and *R. norvegicus* (3UEK)). An additional enzyme that exhibits PARG-like activity is ADP-ribosyl hydrolase ARH3 [48], a 39-kDa protein thought to be confined to the mitochondria [49]. ARH3 is less specific in degrading PAR and is also able to hydrolyse *O*-acetyl-ADP-ribose [50]. *O*-acetyl-ADP-ribose is a specific sirtuin metabolite and has been implicated as a signalling molecule that modulates cellular functions related to NAD-dependent protein deacetylation [51]. Neither PARG nor ARH3 are able to cleave the ester bond between the proximal ADP-ribose unit and acceptor glutamate of target proteins. Although the activity accountable for removing the proximal ADP-ribose unit from the target protein was first detected in human cell extracts and partially purified nearly three decades ago, the identity of the enzyme(s) responsible for the removal of the mono(ADP-ribose) from PARP protein targets remains unknown [52] (Figure 1).

## 2. Structural and Functional Diversity of PAR-Binding Modules

Poly(ADP-ribosyl)ation regulates many important processes and therefore this signal must be recognised in a timely fashion to allow the appropriate functioning of the cell. The recent discovery of protein domains that specifically recognise and bind to different forms of ADP-ribose and/or a part of the PAR polymer, has significantly improved our understanding of the complex roles of poly(ADP-ribosyl)ation signalling. Over the last decade, four evolutionary conserved PAR-binding modules have been discovered within numerous proteins with various functions, providing an insight into the regulation of the cellular processes mediated by poly(ADP-ribosyl)ation.

### 2.1. PAR Binding Motif (PBM)

The PBM was the first discovered protein motif with the ability to recognize and bind PAR [29]. It consists of only 8 amino acids with the following sequence: [HKR]xx[AIQVY]-[KR]-[KR]-[AILV]-[FILPV] [53]. It is loosely defined and therefore it is difficult to predict this motif from genome sequences with a high degree of certainty. The X-ray repair cross-complementing gene 1 (XRCC1) is a prominent example of a DNA repair factor that is recruited to DNA strand breaks, through the interaction of its PBM with the PAR synthesized by DNA damage inducible PARPs. In silico predictions indicate the presence of the PBM in more than 800 proteins. Although the functional role of the PBM in the vast majority of these proteins has yet to be confirmed, the broad presence of this motif implies its general role in PAR mediated processes. Although some PBM containing polypeptides have been crystallized, a crystal structure of the PBM in complex with PAR or a PAR fragment is not available, and therefore the precise molecular nature of this interaction is not clear [54]. However, current data implies that the PBM, through its basic residues, forms an electro-positive surface that non-specifically binds to the negatively charged PAR polymer [55].

### 2.2. Poly(ADP-Ribose)-Binding Zinc Finger (PBZ)

The PBZ was recently discovered to be a specific and high affinity PAR-binding module [40] and possesses the motif consensus sequence [K/R]xxCx[F/Y]GxxCxbbxxxxHxxx[F/Y]xH. The PBZ appears to be much less widespread compared to PBM and is restricted to only eukaryotic proteins (excluding yeast). To date, only three mammalian proteins have been identified that possess the PBZ domain (APLF, CHFR and SNM1A). However, this module appears to be more widely utilised by proteins in other eukaryotes such as the slime mold *Dictyostelium discoideum*, where the PBZ is found in a number of DNA damage response proteins including PARPs, Ku70, Chk2, and Rad17 [40]. In mammals, PAR recognition by the PBZ motifs of CHFR and APLF has been shown to be indispensable for their function in mitosis and DNA repair, respectively [40,56]. The recently solved structures of APLF showed that its tandem PBZ domains could independently bind PAR, but the presence of both functional PBZ domains results in more than a 1000 fold increase in PAR binding affinity [57,58]. The solution structure of the APLF PBZs also revealed that this family of zinc fingers are the most similar in structure to ssRNA-binding C_3_H_1_ Tandem Zinc Fingers (TZF). Both the PBZ and TZF fingers largely lack secondary structure, and the recognition of the substrate is achieved through main-chain-base hydrogen bonding and the interaction with highly conserved aromatic residues [57]. The structures of the APLF and CHFR PBZs suggest that a single PBZ module contains two binding sites that can simultaneously recognize adenines in two neighbouring ADP-ribose units of the PAR polymer [57,59] (Figure 1). This type of recognition makes the PBZ motifs truly specific PAR binding modules.

### 2.3. WWE Domain

The WWE domain, consisting of two conserved tryptophan residues and a glutamate residue, was first noted in proteins related to either ubiquitination or poly(ADP-ribosyl)ation [60]. However, the function of this domain in PAR binding was reported only recently for the RNF146 ubiquitin ligase E3 [61,62,63]. Tandem affinity purification reveals that a number of proteins involved in the DNA damage response, including PARP1, XRCC1, DNA ligase III, KU70, KU86 and histones, are targeted to proteosomal degradation through PAR recognition and their subsequent polyubiquitination by RNF146 [62]. Similarly, the interaction between RNF146 and poly(ADP-ribosyl)ated axin, promotes the degradation of this key regulator of the Wnt signalling pathway [61]. The underlying mechanism of the RNF146 interaction with PAR was recently revealed through the determination of a high resolution crystal structure of the RNF146 WWE domain in complex with iso-ADP-ribose [64]. The molecule of iso-ADP-ribose represents a minimal PAR fragment that contains a specific 2',1''-*O*-glycosidic ribose-ribose bond unique to the PAR polymer (Figure 1). RNF146 does not bind mono(ADP-ribose), but interacts with iso-ADP-ribose with high affinity. The phosphate groups on each side of the iso-ADP-ribose molecule interact with the highly positively charged edge of the WWE domain binding site, while the adenine ring is inserted in the binding pocket formed by the half β-barrel and the α-helix. The mechanisms of recognition explain the specificity of the WWE domain for iso-ADP-ribose and consequently the PAR polymer [64].

### 2.4. Macrodomain

PAR-binding macrodomains are evolutionary conserved structural modules of 130–190 amino acids that are found in proteins with diverse cellular functions (henceforth, in this review the term "macrodomain" refers to PAR-binding macrodomains) [65]. In contrast to the PBZ and WWE domains, the presence of macrodomains is not restricted to eukaryotes but is widespread in all kingdoms of life, including prokaryotes and archaea. Af1521 from a thermophilic archeabacterium was the first macrodomain protein for which the binding of NAD metabolites, including ADP-ribose was reported [66]. The crystal structure of this macrodomain along with additional, more recently solved macrodomain structures of several other proteins, revealed the general structural fold and the ligand binding pocket of this PAR binding module [66,67,68,69,70]. These studies confirmed the presence of high structural homology among macrodomains, which fold into a globular mixed α-helix/β-sheet structure and form a deep L-shaped ligand binding pocket. Despite the high structural conservation, the sequence variation between macrodomains directs their preferences for binding specific forms of ADP-ribose. Histone H2A variant MacroH2A1.1 contains a macrodomain through which it is able to bind PAR and mono(ADP-ribose), as well as *O*-acetyl-ADP-ribose [67,70]. The chromatin remodeler ALC1 uses its macrodomain to bind the PAR synthesized by PARP1 in response to DNA damage [31,71]. Through this mechanism, ALC1 is rapidly recruited to the sites of DNA damage as an important part of the DNA repair machinery. Although the mechanism of PAR recognition by the macrodomain module has not yet been not fully resolved, the crystal structure of the histone MacroH2A1.1 in complex with ADP-ribose suggests that the macrodomain is only able to bind the terminal ADP-ribose units of the PAR polymer [70] (Figure 1). In addition to their binding ability, some macrodomains also exhibit catalytic activity on various ADP-ribose derivatives, making the macrodomains unique among the other PAR-binding modules. The first catalytically active macrodomains were reported for viral macrodomains including the severe acute respiratory syndrome coronavirus (SARS-CoV) and the yeast homolog Poa1p (YBR022). These proteins dephosphorylate ADP-ribose-1″-phosphate, a metabolite that arises from tRNA splicing [72,73,74]. It was also recently demonstrated that several macrodomain containing proteins can deacetylate *O*-acetyl-ADP-ribose *in vitro*. This group comprises the eukaryotic MacroD proteins (orthologues of human MacroD1 and MacroD2 proteins) and a human C6orf130 protein [68,69]. The most recent example of a protein with a catalytically active macrodomain fold is PARG. The first crystal structure of the PARG enzyme revealed that this protein uses a diverged macrodomain for the binding and hydrolysis of PAR [10]. We discuss PARG more extensively in the following chapter. 

## 3. PARG—A New Member of the Macrodomain Family of Proteins

### 3.1. Catalytic Activity of PARG Enzymes

**Figure 2:**
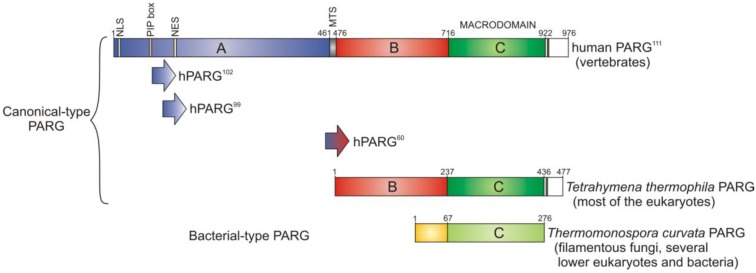
Domain structure of different PARG proteins. The full-length human PARG111 contains an *N*-terminal regulatory region (A-domain; blue) which includes a nuclear localisation signal (NLS), PCNA binding motif (PIP box) and a nuclear export signal (NES). Full length human PARG also contains a mitochondrial targeting sequence (MTS), a highly structured and conserved B-domain (red), and the essential catalytic macrodomain (C-domain; green). Two shorter isoforms of human PARG, PARG102 and PARG99, lack the NLS and PIP box, while PARG60 lacks the entire regulatory region. Human and *Tetrahymena thermophila* PARGs represent canonical-type PARGs and share high conservation in their B-domain and macrodomain. Highly divergent bacterial-type PARG represented by *Thermomonospora curvata* PARG contains the catalytic macrodomain and an *N*-terminal accessory element (yellow).

**Figure 3:**
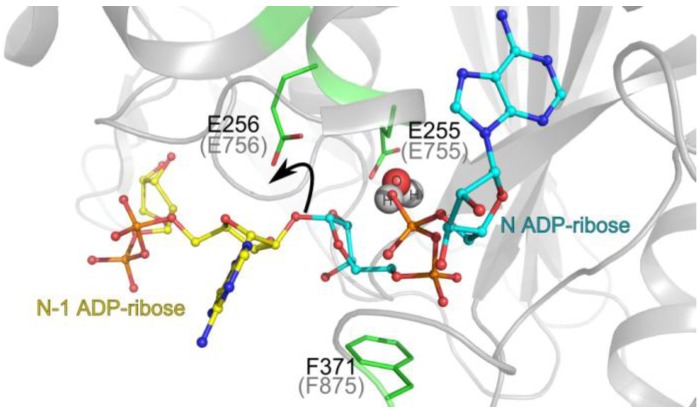
PARG catalytic mechanism. The positions of the reaction product N ADP-ribose (teal backbone; observed in the crystal structure) and a model of N-1 ADP-ribose (yellow) are represented in the *Tetrahymena thermophila* PARG active site. The key *O*-glycosydic ribose-ribose bond is positioned in direct hydrogen bonding contact with the catalytic glutamate 256 (Glu756 in human PARG; grey brackets). The 2’-OH leaving group of n-1 ADP-ribose’ is protonated (black arrow) by the catalytic glutamate. A subsequently formed oxocarbenium intermediate is stabilised by the close proximity of the terminal diphosphate group, which is restrained by the conserved phenylalanine 371 (Phe875 in human PARG). A water molecule (shown in spheres) is ideally positioned to attack this oxocarbenium intermediate. This leads to release of ADP-ribose.

PARG catalyses the hydrolysis of the ribose-ribose bonds of the PAR polymer and is the major enzyme responsible for reversing poly(ADP-ribosyl)ation in cells. Predictably, PARG follows the phylogenetic distribution of PARPs and is found in all eukaryotes, with the exception of yeast, and is also present in a scattering of bacterial species [10]. Filamentous fungi, several other lower eukaryotes and a few bacteria possess a highly divergent form of PARG (which we refer to as bacterial-type PARG) [10], consisting essentially of a macrodomain with a relatively small alpha helical accessory extension at the *N*-terminus (Figure 2). The PARGs in the majority of eukaryotes (ranging from protozoa to humans and referred to as canonical PARGs) are architecturally more complex and their minimal catalytic unit contains an additional highly structured, conserved domain (domain B; *N*-terminal accessory domain) that is amalgamated with the macrodomain (Figure 2) [46,47,75]. Nevertheless, the minimal bacterial PARG architecture has been shown to be sufficient for efficient PAR hydrolysis activity *in vitro* [10]. The function of the *N*-terminal accessory domain in canonical PARGs has not yet been established. However, structural analysis suggests that both the larger canonical PARG *N*-terminal accessory domain and the smaller bacterial alpha-helical extension stabilise the PARG catalytic loop, which is itself a part of the PARG macrodomain. In all PARGs, the macrodomain is located at the C-terminus of the protein (Figure 2) and its catalytic loop contains the consensus signature sequence (GGG-X6-8-QEE,) including two consecutive glutamate residues. Both glutamates are critical for catalysis, and mutation of these residues leads to a complete loss of PARG activity [10,47,76]. A genetic screen in *Arabidopsis thaliana* identified a conserved glycine in the PARG signature sequence as another important residue for PARG function *in vivo* [77]. Structural alignments suggest that the catalytic loop has been evolved specifically by PARGs and is absent in other macrodomain proteins. Conversely, the diphosphate-binding loop, which flanks the opposite side of the ADP-ribose-binding cavity, is highly conserved between PARG and other macrodomains (GVFG motif) [10]. The high structural conservation of the active site suggests that the catalytic mechanism of all PARGs is the same (Figure 3): binding of the terminal ADP-ribose (referred to as N ADP-ribose later in text) positions the unique *O*-glycosydic ribose-ribose bond between N and N-1 ADP-ribose groups in direct hydrogen contact with the two glutamates of the PARG signature sequence. The N-1 ADP-ribose 2’-OH leaving group, attached to the rest of the PAR chain, is protonated by the second glutamate of the PARG signature sequence (Glu256 in *T. thermophila* and Glu756 in human PARGs), forming a positively charged oxocarbenium intermediate. The oxocarbenium intermediate is stabilised by the close proximity of the terminal adenosine diphosphate group, which is appropriately positioned by the restraints imposed by the conserved phenylalanine residue from the macrodomain-wide GVFG motif (Phe371 in *T. thermophila* and Phe875 in human PARG). The unstable oxocarbenium intermediate is then nucleophilically attacked by a water molecule, which is pre-activated by the first glutamate of the PARG signature sequence (Glu255 in *T. thermophila* and Glu755 in human PARG). As a result, mono(ADP-ribose) (N ADP ribose) is released as the main reaction product [10,47]. Despite the uniform catalytic mechanism, it appears that somewhat distinct PAR-binding modes may exist between the canonical and bacterial-type PARGs. While the structural features of the active site, involved in the positioning of the ribose-ribose bond are highly conserved (*i.e.*, the presence and positions of the two consecutive glutamates and the restraints imposed by the phenylalanine residue), there are significant differences in the N ADP-ribose adenosine binding region. In canonical PARGs, a loop named the tyrosine clasp [46] contains a highly conserved tyrosine residue (Tyr296 in *T. thermophila* and Tyr795 in human PARG) which interacts with the adenine base of the central, N ADP-ribose [46,47]. Mutation of this residue moderately affects PARG activity *in vitro* [47]. The equivalent of this loop is not found in bacterial-type PARGs. Despite the additional interaction, the canonical PARG active site is relatively open. In contrast, the bacterial-type PARGs can only bind to the terminal ADP-ribose group of the PAR chain due to the constraints imposed by the C-terminal region and are thus obligate exoglycohydrolases. In comparison, the open active site of canonical PARG might also allow PAR binding in the endoglycohydrolase mode (in addition to the exoglycohydrolase mode ubiquitous in all PARGs). Such endoglycohydrolase activity of the canonical PARGs has never been demonstrated using recombinant PARGs, but it has previously been reported using native PARGs purified from vertebrate cells [78]. The question of how significant is the PARG endoglycohydrolase activity compared to its exoglycohydrolase mode still requires further investigation.

### 3.2. Regulation of PARG Enzymes

Vertebrate PARGs possess an additional *N*-terminal regulatory region which spans approximately 50 kDa (A-domain, residues 1-456 in rat PARG, 1-460 in human PARG), but this domain is not required for PARG catalytic activity *in vitro* [10,75]. The PARG regulatory region is poorly characterised, but it was recently discovered that it bears a PCNA (proliferating cell nuclear antigen) binding motif (PIP box), which was shown to be critical for PARG recruitment to DNA damage sites through a PAR-independent pathway [79]. PCNA binds to DNA and acts as a binding site and processivity factor for numerous proteins involved in DNA replication and repair [80,81]. This additional mechanism of PARG recruitment has most likely evolved to enhance its recruitment efficiency to DNA damage sites. There is a short 16 residue motif which connects the aforementioned regulatory domain and the catalytic region of vertebrate PARGs [46]. This motif bears a sequence resembling the mitochondrial targeting sequence and has been called the MTS motif. However, there is no evidence that PARG, under physiological conditions, is present in the mitochondria. Deletion and site-directed mutagenesis analysis revealed that the MTS of human PARG is important for the catalytic activity and stability of the protein [47,75]. With the exception of vertebrate PARGs, all other canonical PARGs lack this MTS motif and contain only a minimal catalytic region consisting solely of an *N*-terminal accessory domain and a macrodomain (Figure 2). In mammals, PARG is encoded by a single gene but is alternatively spliced into multiple isoforms. The longest isoform PARG111 is targeted to the nucleus due to the presence of a nuclear localisation signal encoded within the first 20 amino acids (Figure 2), whilst the shorter isoforms such as PARG^102^ PARG^99^ and PARG^60^ lack this motif and are consequently cytosolic [82,83].

### 3.3. PARG as a Therapeutic Target

Given the importance of the cellular processes controlled by PARPs, targeting poly(ADP-ribosyl)ation in the therapy of human disease has attracted significant attention over the past several years. It was shown for example that PARP inhibitors can be highly effective against hereditary breast and ovarian cancers [84,85,86]. However, the redundancy of PARP genes with overlapping yet distinct functions makes the targeting of specific PARP dependent pathways difficult. Targeting PARG may represent an alternative approach in modulating poly(ADP-ribosyl)ation. In contrast to PAR synthesis that is mediated by several PARP genes, PAR degradation is predominately carried out by PARG, which is encoded by a single gene. Furthermore, PARG has low cellular abundance, with approximately 2,000 molecules per cell [87]. These properties make PARG an attractive pharmacological target for modulating poly(ADP-ribosyl)ation. Knock down studies using RNAi silencing in human cells have already demonstrated that PARG deficiency results in increased sensitivity to radiation, DNA-alkylating agents and chemotherapeutics, providing indirect evidence of the potential therapeutic relevance of PARG inhibition [88,89,90,91,92]. All existing PARG inhibitors are non cell-permeable and can therefore only be useful for *in vitro* experiments. One such inhibitor is an ADP-ribose analogue, adenosine diphosphate-(hydroxymethyl)-pyrrolidinediol (ADP-HPD) [93]. A more recent example are the small compounds with a rhodanine-scaffold (RBPIs). RBPIs act as specific and easily synthesized *in vitro* PARG inhibitors [94]. It has been suggested that further optimisation of these small molecule inhibitors by applying the structure activity relationship data from the co-crystal structure of canonical PARG in complex with RBPI-3, could significantly improve their efficiency and cell-permeability [47]. In general, the recent progress in understanding the structure and catalytic mechanism of PARGs should enable *in silico* predictions of more potent and specific PARG inhibitors. The development of small, cell-permeable PARG inhibitors would not only provide a major advantage as a powerful tool to study PAR metabolism, but could also potentially become a useful drug for the treatment of human disease.

## 4. Conclusions

Poly(ADP-ribosyl)ation has been recognized as an important post-translational protein modification involved in numerous cellular functions including the DNA repair, transcription and mitosis. The discovery of ADP-ribose binding modules that bind to various forms mono- and poly- ADP-ribose has provided important insights into how ADP-ribosylation regulates different cellular pathways. Among the four distinct PAR-binding modules discovered so far, it is the macrodomain alone that, in addition to possessing binding activity, in some instances also supports a catalytic activity toward ADP-ribose derivates. PARG is the major enzyme involved in the regulation of poly(ADP-ribosyl)ation through the efficient degradation of PAR polymers and this essential catalytic function is mediated largely through PARG’s macrodomain. Whereas our understanding of the PARG catalytic mechanism has been more intensively studied in recent years, we know comparably much less about the physiology and regulation of this enzyme in human cells. We hope that future studies of the PARG enzyme, along with the development of cell-permeable PARG inhibitors will provide new breakthroughs in understanding the cellular pathways regulated by poly(ADP-ribosyl)ation and new ways of exploiting these pathways for the treatment of human disease.
